# Role of tocilizumab for concomitant systemic fungal infection in severe COVID-19 patient: Case report

**DOI:** 10.1097/MD.0000000000025173

**Published:** 2021-03-26

**Authors:** Anggraini Permata Sari, Nikko Darnindro, Aryan Yohanes, Muhammad Ikhsan Mokoagow

**Affiliations:** Department of Internal Medicine, Fatmawati General Hospital, South Jakarta, DKI Jakarta, Indonesia.

**Keywords:** acute respiratory distress syndrome, case report, concomitant candidemia, Coronavirus Disease-19, tocilizumab

## Abstract

**Rationale:**

Bacterial and fungal infections in Coronavirus Disease-19 (COVID-19) patients have been inadequately investigated and reported thus far. The safety profile of tocilizumab (TCZ) administration in candidemia patient still debatable.

**Patient concerns:**

A 54 year-old woman presenting with weakness on the left side of her body was diagnosed with COVID-19. After 7 days of admission, her condition worsened and developed respiratory distress and was having respiratory distress despite standard treatment.

**Diagnoses:**

Acute respiratory distress syndrome (ARDS) in COVID 19 was diagnoses based on real time-PCR swab, deterioration of PaO_2_/FiO_2_ and increased of acute phase reactants.

**Interventions:**

Anti Interleukin–6 (IL-6) was considered to tackle her inflammatory condition. Prior to TCZ administration, blood culture was performed and the result came with *Candida tropicalis* in the absence of bacterial growth.

**Outcomes:**

No major complications associated with intravenous antifungal or TCZ occurred. After 40 days of hospitalization, the patient's clinical condition improved and was finally discharged.

**Lessons:**

This case underscores the safety profile of giving TCZ in candidemia as a secondary infection in severe COVID-19 patient.

## 1 Introduction

The novel Coronavirus Disease-19 (COVID-19) outbreak started in December 2019 in Wuhan, China, and has emerged as a major pandemic.^[1]^ Severe acute respiratory syndrome coronavirus 2 (SARS-CoV-2), an enveloped positive-stranded RNA virus, was later identified as the causative agent.^[2]^ Patient with severe cases develop pneumonia that can lead to Acute respiratory distress syndrome (ARDS).^[3]^ The case fatality rate of COVID-19 has been estimated to 0.2% to 25%, depends on the country.^[1]^ Meanwhile in Indonesia, case fatality rate reached 4,76% by July 2020.^[4]^

ARDS develops in 42% of patients presenting with COVID-19 pneumonia, and 61% to 81% of those requiring intensive care.^[5]^ COVID-19 ARDS appears to have worse outcomes than ARDS than ARDS from other causes. The intensive care unit (ICU) and hospital mortality from typical ARDS are 35.3% (95% CI, 33.3%–37.2%) and 40% (95% CI, 38.1%–42.1%), respectively.^[6]^ ARDS in COVID 19 with concomitant candidemia was never reported, meanwhile the mortality of blood stream candida Sp infections in non COVID-19 population has been reported to be as high as 30^[7]^ and up to 60%.^[8]^

TCZ is one of the immunomodulatory drugs that have been tested in clinical care of the treatment of severe COVID-19 pneumonia.^[9]^ Spinello Antinori and colleagues reported the incidence of candidemia in 3 of the 43 severe COVID-19 patients treated with TCZ. This report has raising the awareness of the risk of candidemia after giving TCZ.^[10]^

## 2 Case report

A 54-year-old woman presented to the emergency department of the Fatmawati General Hospital with weakness on her left side since 5 days before admission. Since her emergency warning system for COVID-19 was highly suspicious, a pharyngeal swab was taken and real-time PCR was positive for SARS-CoV-2, confirming COVID-19. She had been taking azithromycin, enoxaparin and an antiviral drug (oseltamivir) for 7 days since admission, with an improvement on her left-side weakness. Her medical history included hypertension and well-controlled type 2 diabetes mellitus with oral anti-diabetic agents. On her 8th day, she developed shortness of breath and arterial blood gases analysis revealed acute hypoxemic (type I) respiratory failure. Multiple diffuse infiltrates on both lungs were apparent on her repeat chest X-ray.

The patient was subsequently put on high-flow oxygen (Flow 50% Fraction 70%) due to respiratory deterioration, fulfilling the criteria for moderate ARDS. Arterial blood gas analysis revealed a pH of 7.49, pCO2 of 34, pO2 of 56,3 and SaO2 of 91,7%. Due to full occupancy of ICU, she was transferred to High Care Unit awaiting availability a non-invasive respiratory support. Serial chest X-ray revealed worsening on both lungs, infiltrates visible as bilateral hazy opacities suggesting possible multifocal pneumonia due to ARDS. Laboratory results revealed elevated acute phase reactants (C-Reactive protein (CRP) 5.9 mg/dl, ferritin 1269 ng/ml, D-dimer 18547 ng/ml, lactate dehydrogenase 781 U/L and lymphopenia (4%)).

**Figure 1:**
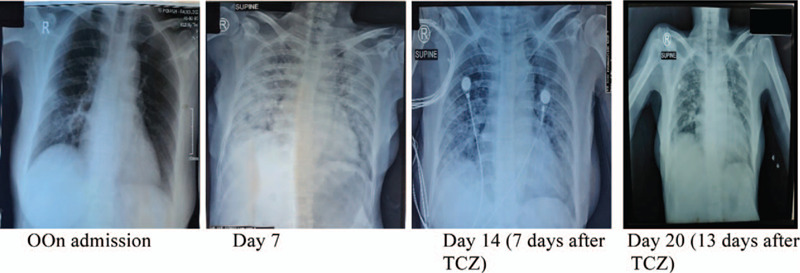
Serial chest x-rays. TCZ was administered at day 7 (when ARDS is confirmed).

Her medications included TCZ, a broad-spectrum antibiotic (meropenem), enoxaparin, intravenous vitamin C, low dose intravenous hydrocortisone and standard care for critically ill patients. Additionally, the patient received 400 mg of TCZ (bodyweight 52 kg). Blood culture sample was withdrawn prior to meropenem and TCZ administration. Three days after TCZ, the blood culture revealed positive for *Candida tropicalis.* Intravenous micafungin was given for 21 days until the blood culture is negative Fig. 1.

During hospitalization, the patient remained stable on high-flow oxygen, and did not show any sign of deterioration due to candidemia, whilst TCZ has been given. Her chest X-ray also showed improvement within days. After 25 days of hospitalization, RT-PCR examination for SARS-CoV-2 became negative Table 1.

**Table 1:** Lymphocyte count, CRP, and oxygen support after giving TCZ.

Day	–1	1	2	3	4	5	6	7
Lymphocyte count (%)	4	6	6	7	10	13	19	19
CRP (mg/dl)	5.9	4.2	2.3	1	0.7	0.7	0.7	0.7
High-flow oxygen (Flow (lpm), O_2_ Fraction %))	50, 70	50, 70	50, 70	50, 70	50, 70	50, 70	Non rebreathing mask (NRM) 12 lpm	Nasal canule 5 lpm

## 3 Discussion

The natural history of severe COVID-19 is thought to be driven by a so-called cytokine storm. The inflammatory cytokine storm observed in COVID-19 shares features with macrophage activation syndrome and hemophagocytic lympho-histiocytosis. In COVID-19, the increases in ferritin are not as high, and organ damage is primarily restricted to lungs.^[3,11–12]^ Importantly, not all patients with COVID-19 undergo cytokine storm, this phenomenon only occurs in a certain subset of severe cases.^[13]^ The classic clinical picture of a patient with cytokine storm involves rapid respiratory deterioration.^[14]^ Our patient began to decline and demonstrated symptoms of respiratory disease after 7 days of admission.

Zhang at al reported that the number of T lymphocytes including both CD4 and CD8 subtypes and especially NK cells are much lower than expected in patients with severe disease course.^[15]^ The number of regulatory T cells is also very low. Severe lymphopenia is a very early sign of the disease, preceding pulmonary problems, and tends to normalize as the patient improves.^[16]^ On the other hand, monocytes and macrophages are increased, which may explain elevated levels of pro-inflammatory cytokines such as interleukin (IL)-6, IL-1, tumor necrosis factor (TNF)-α, and IL-8.^[17]^

TCZ is a recombinant humanized anti-human Interleukin–6 (IL-6) receptor monoclonal antibody, preventing IL-6 binding to its receptor to exert the immunosuppression promoted by IL-6. Recently, an observational cohort study from Italy found TCZ might reduce the risk of invasive mechanical ventilation or death in patient with severe COVID-19 (Lancet Italy). Another study involved 63 patients with severe COVID-19, and TCZ succeeded in improving respiratory and laboratory parameters, such as Pa0_2_, Fi0_2_, consequently and increased the likelihood of survival.^[18]^

A study from Milan, Italy reported 43 patients with severe COVID-19 pneumonia were treated with TCZ and 3 patients (6.9%) developed candidemia, 1 with endophthalmitis and endocarditis. Only one of the 3 patients had been previously hospitalized in the ICU and, at the time of diagnosis of candidemia, only one had a central venous line on site and his blood infection can be considered catheter related. All the patients had received parenteral nutrition during hospitalization and 2 had been treated with antibiotics.^[10]^

Another study assumed the high prevalence of candidemia among patients treated with TCZ can be the consequence of multiple well known risk factors it can be speculated that the suppression of IL-6 response might contribute to this blood infection.^[19]^ Interestingly, previous studies conducted in interleukin-6 deficient mice showed that they were more susceptible to systemic *Candida albicans* infection, had a decreased survival and an increased fungal load in their organs when compared with IL-6 positive controls.^[20,21]^

In our patient, we obtained the blood culture sample before TCZ was administered, implying that the patient's candidemia is unlikely due to TCZ. Moreover, the patient did not receive parenteral nutrition prior to TCZ use and only treated with oral azithromycin.

Our case highlights the safety profile of giving TCZ in a patient with an existing candidemia. Our clinical observation did not demonstrate any worsening symptoms of candidemia. After 21 days of treatment with mycafungin, the blood culture reported negative result. Therefore, although reports to date are anecdotal, there may be a role for giving TCZ in fungal infection patient, as long as clinically indicated.

## Author contributions

**Conceptualization:** Anggraini Permata Sari.

**Investigation:** Anggraini Permata Sari.

**Resources:** Anggraini Permata Sari.

**Validation:** Anggraini Permata Sari.

**Visualization:** Anggraini Permata Sari.

**Writing – original draft:** Nikko Darnindro, Aryan Yohanes, Muhammad Ikhsan Mokoagow.

**Writing – review & editing:** Anggraini Permata Sari.
